# Co-development and implementation of a group-based arm-crank exercise programme in the community for individuals with neurological impairments

**DOI:** 10.1186/s13102-025-01507-6

**Published:** 2026-01-27

**Authors:** Shin-Yi Chiou, Millie Taylor, Ruo-Yan Wu, Joshua Kearney, Maria del Rocio Hidalgo Mas, Prerna Mathur, Tom E Nightingale

**Affiliations:** 1 School of Sport, Exercise and Rehabilitation Sciences, College of Life and Environmental Sciences, University of Birmingham, Edgbaston, Birmingham, B15 2TT United Kingdom; 2 International Collaboration on Repair Discoveries, Faculty of Medicine, University of British Columbia (UBC), British Columbia (BC), Vancouver, Canada

**Keywords:** Group-based delivery, Disability, Rehabilitation, Upper-body exercise, Inclusive exercise, Health, Function

## Abstract

**Background:**

Group-based exercise classes are popular ways to promote engagement in regular exercise. However, such opportunities are limited for individuals with neurological impairments, who often are more physically deconditioned, require disability-specific instructions and specialised, accessible equipment.

**Objective:**

This study aimed to co-develop a group-based, community-delivered arm-crank exercise (ACE) programme with individuals with neurological impairments and to evaluate the feasibility of implementing the programme in community settings.

**Methods:**

A pragmatic ACE programme was developed with five participants with a long-term neurological impairment, followed by a pilot, implementation of the programme in a university-based wellbeing centre (MoveWell) and in a local gym (Greenbank) across multiple iterations. The classes were held twice a week for 8–12 weeks, guided by music and real-time heart rate monitoring to maintain moderate-to-vigorous intensity exercise. A mixed-methods evaluation assessed adherence, participant satisfaction, health-related quality of life (HRQoL), and physical function. Focus groups were conducted to explore perceived benefits, challenges, and recommendations for future implementation.

**Results:**

Ten participants (mean ± SD age 45 ± 14 years; 2 females) with diverse neurological impairments (spinal cord injury, stroke, hereditary spastic paraplegia, cerebral palsy, and Chiari malformation) completed a minimum of one iteration of the programme. Adherence was high (MoveWell: 77 ± 17%; Greenbank: 54 ± 7%) and no serious adverse effects were reported. Participants reported increased of 10 points (SD = 12) in both physical and mental component summaries of HRQoL, with high self-perceived satisfaction and effectiveness with the programme. Qualitative data highlighted that self-perceived physical and mental benefits, social connection, and accessibility as key facilitators for engagement.

**Conclusion:**

The programme, co-developed with the participants, was feasible, acceptable, and safely delivered in real-world community settings. Findings support the potential for inclusive, group-based ACE to promote health and wellbeing in people with neurological conditions and inform future community-based exercise initiatives.

**Supplementary Information:**

The online version contains supplementary material available at 10.1186/s13102-025-01507-6.

## Background

Exercise is a critical component of a healthy lifestyle for all individuals, including those living with neurological conditions and mobility impairments. Evidence consistently shows that regular exercise improves physical function, psychological wellbeing, and the management of secondary chronic conditions in this population [1–3]. Despite these benefits, individuals with neurological impairments participate in exercise at lower rates than the general population [4, 5].

To address this, exercise guidelines for health have been developed for people with neurological impairments [6–8]. However, people with neurological impairments generally less likely to meet the guidelines and are at higher risk of developing long-term conditions related to inactivity than people without an impairment or disability [4]. Commonly cited barriers include health concerns, high costs of equipment or programmes, pain, limited access to accessible facilities, lack of time, and fear that exercise may exacerbate symptoms [9–11]. These challenges are particularly pronounced for individuals who rely on wheelchairs for mobility. Group-based exercise has been shown to promote motivation and adherence to exercise engagement in the general population [12–14] such opportunities are limited in community settings for people with neurological impairments.

Arm-crank exercise (ACE), which involves upper-body aerobic exercise using an arm-crank ergometer, offers a usable exercise modality for individuals who cannot engage in lower-limb or full-body exercise. Studies have demonstrated that ACE can improve cardiorespiratory fitness, upper-body strength, seated balance, and health-related quality of life (HRQoL) [15–18]. However, ACE remains largely confined to clinical or research settings. This may be due in part to the high cost of ergometers and the absence of established group-based ACE classes, which are common for lower-body cycling (e.g., gym-based ‘spin’ classes).

Moreover, people with neurological impairments often have diverse symptoms and levels of ability, contributing to perceptions that group exercise classes are infeasible due to the need for highly individualised prescriptions or unsafe. This medicalised view of disability has also contributed to structural exclusion, with few community exercise options being made accessible to people with neurological conditions. This gap reflects broader disparities in access to health-promoting environments and services. Concerns about safety, such as exercise-induced fatigue, high blood pressure, or spasms, further limit implementation. While studies have shown that exercise can alleviate such symptoms, most of this research has taken place in controlled environments and performed with real-time physiological monitoring which is not always feasible in community settings.

Recent advances in wearable technologies have improved the feasibility of monitoring exercise intensity outside laboratory or clinical environment [19, 20]. Devices such as chest-worn heart rate monitors (e.g., Polar H10) are used in research due to their accuracy and are also commercially available for use in community and fitness settings. Portable blood pressure cuffs are widely available in gyms and leisure centres. This can facilitate the safe translation of lab-based exercise protocols into real-world contexts. However, there remains limited evidence on how acceptable and feasible it is for people with neurological impairments to engage in such monitored exercise programmes outside of clinical settings.

Hence, this study had two aims. First, to develop with the users an evidence-informed, group-based ACE programme tailored for individuals with neurological and lower-limb impairments. Second, to implement and evaluate the programme in two real-world community settings, a university-based community wellbeing centre and a local gym, assessing its feasibility, acceptability, and perceived benefits. This study builds on existing laboratory research to test a scalable and inclusive model that enables participants to exercise at intensities aligned with health guidelines. It also addresses the lack of accessible group-based programmes in the community and explores how such models could be embedded into broader community-based healthcare provision.

## Methods

### Recruitment and screening

**Fig. 1:**
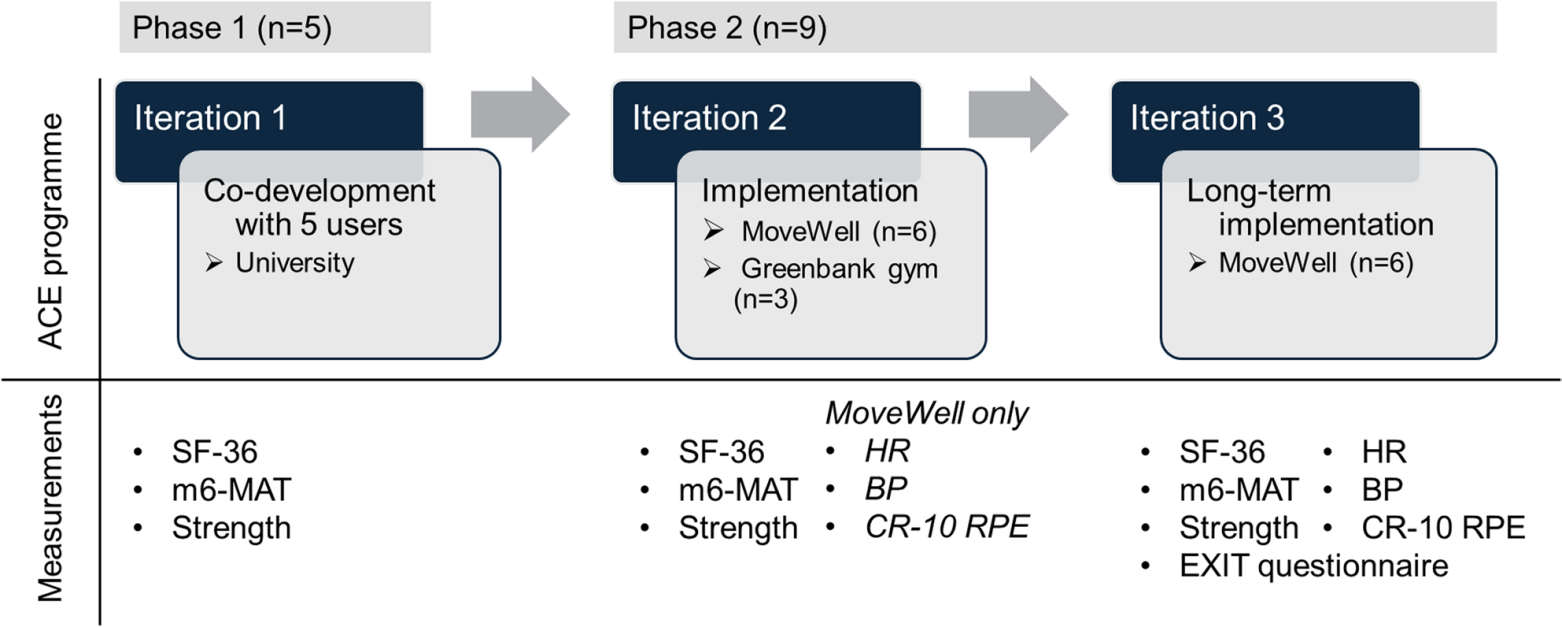
Study diagram. ACE: arm-crank exercise; SF36: short-form 36; m6-MAT: modified 6-Minute Arm Test; HR: heart rate; BP: blood pressure; RPE: rating of perceived exertion. N: number of participants

The study protocol was approved by the University of Birmingham STEM ethics committee (ERN_1200), and all procedures were performed in accordance with the declaration of Helsinki. Recruitment took place via research newsletters, social media, and communication with clients at the host sites. Adults (≥ 18 years) with a neurological impairment resulting in limited lower-limb mobility who were able to follow exercise instructions and demonstrated sufficient upper-body function to perform arm cycling were deemed eligible. Individuals were excluded if they had ongoing shoulder injuries or contraindications to physical activity, as assessed by the Physical Activity Readiness Questionnaire (PAR-Q). All participants provided written informed consent. A study diagram is presented in Fig. 1.

### Phase 1 – programme co-development

Phase 1 ran from June to July 2024. Several core components were pre-defined for the development of the arm-crank interval exercise programme: [1] it targeted adults with neurological impairments and limited lower-limb mobility who use a wheelchair as their primary mobility aid [2], it utilised equipment that was wheelchair accessible [3], it focused on upper-body and trunk movement, and [4] it was inclusive of individuals with varying fitness levels. These components were chosen to ensure the programme would be beneficial to individuals unable to perform lower- or full-body exercises and could be easily adopted by individuals or health and wellbeing organisations.

Drawing from the literature and our previous research [15, 16, 21], we developed a group-based spin class using an arm-crank ergometer. Each class lasted approximately 50 min and included a warm-up, ~ 12 bouts of interval exercise at moderate-to-vigorous intensity (target heart rate [HR] ranging from 64% to 95% of maximum HR) [22], followed by a cool-down and stretches. Evidence has shown that exercise to the music has positive impact on psychophysiological responses, such as increased exercise enjoyment and exercise duration, and reduced rating of perceived exertion (RPE) [23–25]. It is suggested that fast-tempo music enhances arousal levels and motivation, while slow-tempo music aids in relaxation [24, 26]. Therefore, resistance, cadence, and/or seated posture were manipulated at the start of each bout to match the tempo of selected music tracks. Exercise intensity was guided using the Borg CR10 scale, with participants instructed to achieve a session CR-10 RPE at 7 or above [27]. At the end of each session, participants were asked “*How hard did the exercise session feel overall?*” and provided their session RPE.

To accommodate varying trunk and upper limb function, resistance and seated posture (e.g. seated with or without back support) were only changed between bouts; cadence was adjusted during each bout to achieve the format of interval training. To minimise the risk of shoulder injury associated with ACE, each arm ergometer was placed on a height-adjustable table.

After the initial design of the programme, we invited 5 volunteers with chronic neurological impairments (P1-P5, Table 1) to take part in the first iteration. Participants underwent two classes a week at University of Birmingham and completed one home-based session per week using pre-recorded 30-minute exercise videos on a day of their choice when no in-person class was scheduled. Each participant was provided with an arm-crank ergometer for home use (MagneTrainer ER). Adherence to the home sessions was confirmed verbally during the in-person classes and recorded by the research team on a spreadsheet. No wearable sensors were used during home sessions. Participants were invited to provide feedback on the programme structure and delivery after each class. We considered and implemented the feedback in next classes. The cycle continued until all the participants were satisfied with the programme.

**Table 1:** Demographic characteristics

Study ID	Age	Sex	Condition	Type of wheelchair used	Participation duration (iteration)	Venue
P1	50	M	C6 complete SCI	Manual	30 weeks (1–3)	MoveWell
P2	37	M	T4 incomplete SCI	Manual	30 weeks (1–3)	MoveWell
P3	29	F	Cerebral Palsy	Power	30 weeks (1–3)	MoveWell
P4	45	F	T10 complete SCI	Manual	30 weeks (1–3)	MoveWell
P5	25	M	C5 complete SCI	Manual	8 weeks* (1)	MoveWell
P6	58	M	Stroke	Manual	22 weeks (2–3)	MoveWell
P7	60	M	Stroke	Walking aid	22 weeks (2–3)	MoveWell
P8	38	M	T6 complete SCI	Manual	8 weeks (2)	Greenbank
P9	69	M	Chiari malformation	Power	8 weeks (2)	Greenbank
P10	43	M	HSP	Manual	8 weeks (2)	Greenbank

M: male; F: female; SCI: spinal cord injury; HSP: Hereditary spastic paraplegia

*Participant enrolled in another research study involving arm-crank exercise after completion of the iteration 1 and therefore became ineligible for the study

### Phase 2 - programme piloting

### Study setting

The programme was piloted at a community centre (MoveWell) at the University of Birmingham (October–December 2024, January–April 2025) and at the Greenbank Sports Academy, a local gym, in Liverpool (October–December 2024).

### Arm-crank spin class

The programme was reported following the Consensus on Exercise Reporting Template checklist. Participants attended twice-weekly arm-crank spin classes for 8 to 12 weeks. A minimum of 8-week duration was chosen based on previous literature [15], with longer periods permitted when resources allowed. Each class was led by 1–2 trained staff or student volunteers from physiotherapy or sport and exercise science programmes at the University of Birmingham. Volunteers received a 90-minute structured training session and instructional materials, as well as ongoing feedback and coaching from the research team.

A publicly available video showing sample classes is included in Supplementary Material 1. Each class followed a structured format (Supplementary Material 2) where the instructor announced upcoming changes in resistance, posture, or cadence in advance, allowing participants time to prepare. Class leaders also assisted participants in adjusting resistance settings and monitored exercise intensity when needed (e.g., for participants with impaired grip function). A chest-worn heart rate monitor (Polar H10) was provided to participants at MoveWell from October 2024 to April 2025 (iteration 2 & 3) but was not expanded to the other site due to equipment availability. Peak heart rate (HR_peak_) was determined using age estimation for most participants and using cardiopulmonary exercise test (CPET) for two participants (P1 & P2) who had impaired autonomic function (e.g., a SCI at T6 or a higher level). The detail of the CPET is described in Supplementary Material 3. Each participant’s HR_peak_ value was entered into the Polar Team application (app), which then displayed real-time HR as a percentage of HR_peak_. Session HR was recorded from the interval exercise and cool-down. The app allowed class leaders to view the HR of all participants simultaneously on a single screen, enabling continuous monitoring without the need to check individual devices. Class leaders used the real-time HR display to provide tailored instructions to each participant during exercise, ensuring that everyone achieved the prescribed exercise intensity (i.e., > 64%HR_peak_).

Blood pressure was taken before and after the class; participants reported their average session CR-10 RPE and any adverse effects (e.g. musculoskeletal pain, skin blisters, or spasms) to the class leaders at the end of each class. They also completed an anonymous class satisfaction rating (1 to 5 stars; 1 = extremely dissatisfied, 5 = extremely satisfied) from January – April 2025) using an online Microsoft form.

### Outcome measures

#### Participant feedback (EXIT Questionnaire)

At programme end (April 2025), participants completed an EXIT questionnaire in which overall satisfaction, perceived benefits, and programme feasibility were each rated on a 0–10 scale (0 = worst; 10 = best). Additional questions addressed motivations, barriers, likelihood of continuation, and preferred frequency of ongoing participation. Participants could also provide open-ended feedback.

#### Class satisfaction ratings

Class satisfaction data were exported from the Microsoft Form, and a mean rating across all available responses was calculated.

#### Health-Related quality of life (HRQoL)

HRQoL was assessed using the short-form 36 (SF-36) questionnaire or the walk-wheel version for non-ambulatory participants. Scores were scaled from 0 (worst) to 100 (best) and grouped into eight subscales: four physical (Physical Component Summary; PCS) and four mental (Mental Component Summary; MCS) subscales.

#### Focus group interviews

Two semi-structured focus groups were conducted post-programme with 6 participants (3 from the MoveWell, and 3 from the Greenbank gym). The focus groups were led by a trained researcher and conducted via Zoom for the participants from MoveWell and in-person for those from the Greenbank. The discussions covered experiences, content, challenges, and broader implementation (Supplementary Material 4). The discussions were recorded and transcribed verbatim.

#### Physical function

Physical function was assessed before and after the programme using a modified 6-Minute Arm-Crank Test (m6-MAT) and maximal voluntary isometric strength.

Due to equipment constraints and variations in functional ability between participants, resistance for the m6-MAT was set to a level that participants could cycle at 60 revolutions per minute (rpm) comfortably; if they struggled to cycle at 60 rpm, resistance was set to the lowest level. Participants were instructed to crank as fast as possible for 6 min. Total distance (as displayed on the device) was recorded; RPE at the first 30 s and immediately after the completion was recorded. The same bike, set at identical resistance, and with the same seating arrangement were used for the pre- and post-assessment in each iteration.

Maximal voluntary isometric strength was assessed for shoulder flexors and extensors of the dominant/less affected arm, and trunk flexors and extensors using a handheld dynamometer (NOD; OT Bioelettronica, Turin, Italy) at MoveWell. Each muscle group was tested twice, with a 60-second rest interval. The highest recorded value was retained.

For shoulder testing, participants sat with their back supported, the shoulder flexed at 90° and forearm in the neutral position. The dynamometer was placed just above the elbow joint. Participants were instructed to flex and extend the shoulder against the resistance provided by the researcher. For trunk testing, participants sat upright without back support, arms resting on thighs, with the dynamometer placed on the sternum (flexors) or the T6 spinous process (extensors). Participants were instructed to flex or extend the trunk against the resistance applied to them by the researcher.

The hand-held dynamometer was not available at Greenbank; therefore, shoulder strength was assessed using repetition maximum testing with a TechnoGym Kinesis One cable machine. Participants performed unilateral, seated shoulder flexion and extension with both the left and right arms to determine the maximum weight they could lift for 5 to 10 repetitions. A two-minute rest was provided between attempts, with the resistance increased by a fixed increment (5 kg) until failure. The higher value between the left and right arms was used to estimate the one-repetition maximum using standard prediction equations [28]. Strength of the trunk was not assessed in the participants from the Greenbank.

Verbal encouragement was provided and kept consistent throughout the assessment. The choice of assessing the strength of the shoulder and the trunk was due to the following reasons: (1) all participants, regardless the type of their injuries, had intact innervation to the deltoid, and (2) prior evidence of improved trunk control via ACE [16, 17].

### Data analysis

Quantitative data were analysed using IBM SPSS v29. Descriptive statistics (mean or median, standard deviation or range) were reported for demographics, physical outcomes, and survey responses. Since the physical outcomes were assessed across different sites, iterations, and equipment types, and participants presented with diverse conditions and baseline abilities, post-assessment values were normalised to pre-assessment values and expressed as percentages relative to pre-assessment to account for this variability. Qualitative data were coded using Microsoft Excel and analysed using a deductive thematic analysis approach [29, 30] to obtain qualitative data related to the discussion themes. Thematic maps were produced to visualise findings.

## Results

### Participant characteristics

Demographics of the participants are detailed in Table 1. From June to July 2024 (1st iteration), five participants were involved in Phase 1. Of those, four continued to participate in the programme from October to December 2024 (2nd iteration), with two additional participants joining in, at the Movewell. During the same period, three participants underwent the programme at the Greenbank gym in Liverpool. All 6 participants in Birmingham continued with the programme from January to April 2025 (3rd iteration). Overall, a total of 10 participants (8 male; mean age 45 years old (SD: 14; range 25–69) enrolled in the programme from June 2024 to April 2025. Main causes of the impairments were SCI (*n* = 5), stroke (*n* = 2), hereditary spastic paraplegia (*n* = 1), cerebral palsy (*n* = 1), and Chiari malformation (*n* = 1).

### Modifications to the programme in phase 1

Several modifications were made to the structure and delivery of the programme in response to participant feedback during Phase 1. Initially, the sessions followed protocols commonly used in ACE studies, prescribing a base cadence of 50–60 rpm. However, this fixed cadence proved challenging for some participants due to varying levels of physical fitness and impairments. Participants suggested adopting a self-selected base cadence to accommodate individual differences. This feedback led to a revised protocol in which participants determined their own base and fast cadences by observing their rpm on the ergometer screen and used these personalised targets during the interval training sessions.

Participants also noted difficulties in following music tracks with rapid or frequent tempo changes (e.g., attempting to accelerate within 10-second segments). Those with more severe impairments found it particularly challenging to adjust their cadence in time with abrupt changes in rhythm. Given that higher cadences are important for eliciting cardiorespiratory benefits, the music was carefully selected, enabling all participants to engage effectively at higher intensities.

Additional modifications included the incorporation of backward pedalling during the cool-down phase, to promote balanced activation of anterior and posterior upper-body muscles. We also revised the class duration protocol to account for progression. Rather than starting with a fixed 50-minute session, we implemented a progressive approach: sessions began at 30 min and increased by 5 min each week (through the addition of an interval bout), until reaching 50 min by the end of the programme. This co-developed ACE programme was then implemented at the MoveWell and the Greenbank gym in Phase 2.

### Results from phase 2 – HR, RPE, and BP during classes

**Fig. 2:**
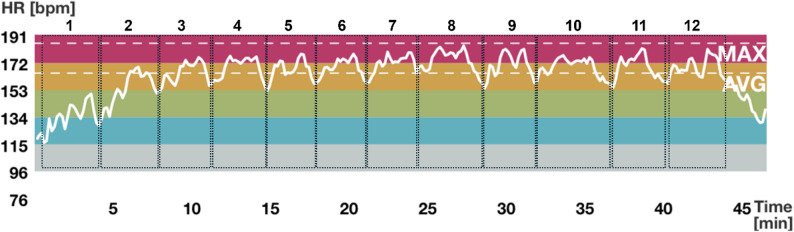
Heart rate (HR) trace from a representative participant during an arm-crank exercise class, recorded using the Polar Team application. The participant’s maximum HR and average HR of the session was 185 beats per minute (bpm) and 164 bpm, respectively. Colour bands represent five HR zones defined by the manufacturer. Vertical axis indicating the HR range; horizontal axis indicating recording time. single rectangle with dotted lines indicating a bout of interval exercise; there are 12 bouts of interval exercise in the session. Cool-down is after the 12th bout of interval exercise

Figure 2 illustrates changing HR across a session from a representative participant; the figure is generated from the Polar Team app. The recording includes 12 bouts of interval exercise, and the cool-down. Note that the HR stays mostly between 153 and 191 bpm, which is 80–100% HR_peak_ for this participant. The HR decreases during the cool-down period.

Individual HR and RPE are presented in Table 2. Group data revealed that participants reached to an average HR of 73 ± 5%HR_peak,_ and 75 ± 4%HR_peak_ during exercise in the second and third iterations, respectively, confirming that participants exercised at moderate-to-vigorous intensity. The average CR-10 RPE of a class was 8 (range: 6–8) and 7 [6–8] in the 2nd and 3rd iterations, respectively (Table 2). Furthermore, the systolic and diastolic BP values before the start of the warm-up were 132 ± 20mmHg and 87 ± 10 mmHg, respectively. The systolic and diastolic BP values after cool-down were 129 ± 11 mmHg and 84 ± 5 mmHg, respectively.

**Table 2:** Average exercise heart rate (HR) across all classes in iterations 2 and 3

	Iteration 2			Iteration 3		
Study ID	**Average HR (%HR**_**peak**_ **)**	**Maximum HR (%HR**_**peak**_ **)**	**Average session CR-10 RPE**	**Average HR (%HR**_**peak**_ **)**	**Maximum HR (%HR**_**peak**_ **)**	**Average session CR-10 RPE**
P1	70 ± 4	90 ± 7	7 (6–8)	72 ± 4	91 ± 11	7 (6–8)
P2	76 ± 6	94 ± 4	8 (7–8)	75 ± 5	91 ± 4	6 (6–8)
P3	75 ± 5	88 ± 6	8 (6–8)	82 ± 3	95 ± 3	8 (7–9)
P4	78 ± 5	93 ± 4	8 (6–8)	78 ± 6	92 ± 6	8 (6–8)
P6	67 ± 1	86 ± 6	8 (6–9)	68 ± 8	76 ± 5	7 (6–8)
P7*	62 ± 8	77 ± 9	8 (6–8)	62 ± 7	78 ± 6	7 (6–9)
Mean	**72 ± 4**	**90 ± 4**	**8 (6–9)**	**75 ± 5**	**88 ± 5**	**7 (6–9)**

Average and maximum HR are presented as mean ± standard deviation; average session CR-10 RPE are presented as median (range). RPE: rating of perceived exertion; HR_peak_: peak heart rate. * P7 being on beta-blocker medication for heart rate control

### Adherence and class satisfaction ratings

There were 22 classes each offered in the second and third iterations, at MoveWell. There were 16 classes offered at Greenbank. Class attendance rates at MoveWell were 82 ± 11%, 72 ± 22% for the 2nd and 3rd iterations, respectively. The adherence to the home sessions was 54% ± 7% from the 2nd iteration; the home sessions became optional in the 3rd iteration and adherence was not recorded. The average class satisfaction rating collected from January to April 2025 at MoveWell was 4.8 out of 5. Furthermore, the attendance rate at Greenbank was 48 ± 20%.

### Self-perceived satisfaction with the programme

Participants rated 10 out of 10 for the overall satisfaction to the programme, and 10 out of 10 for the benefits from the programme. Participants rated 8.5 out of 10 (range: 6–10) for the feasibility of fitting the programme in their routine. Regarding the enjoyment of the programme, 90% indicated the classes being fun to do and a sense of achievement after the class, followed by 85% indicating the class energising and uplifting spirit. 80% of the participants enjoyed meeting and having conversations with other people in the class. Barriers that limited participation in the programme were: can’t find time to attend the class (29%), arrangement for travel and carer to come to the class (25%), and illness (e.g., bowel issues; 17%). Furthermore, 83% of the participants agreed that the programme is a good way to improve/maintain fitness and would continue the programme two times a week. The free text feedback demonstrated a broader physical benefit from the programme, including improved strength and mobility, pain reduction, better bowel and bladder function and skin integrity, weight loss, better sleep quality, and improved mood and mental health.

### Adverse events

Two participants reported mild skin irritation caused by friction between the skin and the pedal of the arm bike during a single session. This was successfully managed by providing exercise gloves and applying surgical tapes to protect the affected areas. One participant reported shoulder pain, although it was noted that the pain may have been associated with playing wheelchair basketball rather than the arm-crank programme. The participant continued with the programme and reported no worsening of symptoms. Furthermore, one participant presented with elevated blood pressure upon arrival at a class, likely due to the physical exertion of using public transportation and a manual wheelchair. The blood pressure returned to the participant’s normal range after a period of rest. No other adverse events were reported.

### Qualitative focus groups

Six participants (4 men and 2 women, aged 29–69 years) attended the focus groups. The presented themes reflect the thematic structure of the interviews: experience of the programme, content of the programme, challenges to participation in the programme, and programme implementation in practice. Sample quotes are in Supplementary Material 5.

#### Theme 1 – experience of the programme

All focus group participants had a positive experience of the programme. Several factors contributed to the positive experience. All participants mentioned social aspects and working out in a group as being an important part of the programme. Participants enjoyed the programme and liked the routine of the programme. Participants were positive about the venues being in the community, rather than in a hospital. Observing noticeable physical and mental benefits from the programme also contributed to their positive experience of the programme.

#### Theme 2 – Programme design and delivery

Participants found exercising at a higher cadence was easier than at a higher resistance. By alternating the cadence and resistance participants felt there was a good balance in the level of challenge during each class. Participants liked exercising to the music, which motivated them as well as the motivation provided by the class leaders, getting to know the class leaders, and the knowledge of the class leaders to their conditions. They also liked the format of the class being like a spin class and the accessibility of the exercise, thereby no need for additional carer or support to exercise. Furthermore, participants liked having real-time heart rate monitor feedback during exercise, so they had an awareness of the relative exercise intensity they were training at.

#### Theme 3 – Challenges to programme participation

Participants expressed less desired for the home-based sessions due to personal and environmental factors, including no suitable table at home to place the bike on to exercise, difficulties in fitting the exercise around house chores or completing a session without distractions. A participant described logistical difficulties to exercise with the pre-recorded videos due to poor hand function and unable to pause the video by themselves.

Furthermore, participants mentioned the schedules of the classes clashed with their work timetables. Some mentioned the heavy traffic in the city during the time when the classes were scheduled affected their participation in the programme. The programme appeared to be difficult for some participants with a lower fitness level at the beginning, which was also mentioned as a barrier.

#### Theme 4 – implementation

All participants expressed the desire for the programme to be in hybrid, that is to have both in-person and online live classes to provide flexibility for scheduling. All participants agreed that the programme is suitable for individuals with a wide range of conditions, extending beyond wheelchair users, and felt that community centres are better placed than clinical settings to deliver such programmes. Participants remarked that inclusive, group-based exercise opportunities tailored to people with mobility impairments are extremely limited in community settings, underlining the perceived value and need for this type of initiative. Some also suggested that the programme could benefit individuals involved in competitive sports, such as adaptive rowers and boxers, as well as young people born with disabilities.

### Health-related quality of life, physical performance and strength

**Fig. 3:**
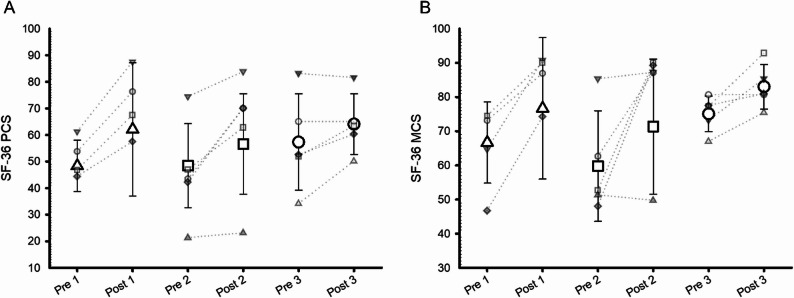
Short Form-36 (SF-36) scores across programme iterations. **A **Physical Component Summary (PCS) and **(B)** Mental Component Summary (MCS) scores from participants who completed two or three programme iterations. Mean values at pre- and post-assessment for the 1st, 2nd, and 3rd iterations are shown using unfilled triangles, squares, and circles, respectively. Individual participant data are shown with smaller symbols, with the same symbol used for each participant across iterations. Error bars represent standard deviations of the mean

The number of participants completed the SF-36 walk-wheel at pre- and post-assessment varied across iterations: 5 completed in the 1st iteration, 8 in the 2nd iteration, and 5 in the 3rd iteration. Therefore, data from each iteration were presented separately. The PCS scores increased by 14 ± 16 points, 9 ± 13 points, and 7 ± 7 points in the 1st, 2nd and 3rd iterations, respectively (Fig. 3A). Additionally, 4 participants improved > 4 points in each iteration, exceeding the minimum clinically important difference (MCID) in PCS [31]. Furthermore, the MCS scores increased by 10 ± 25 points, 12 ± 18 points, and 8 ± 6 points, in the 1st, 2nd and 3rd iterations, respectively (Fig. 3B). The number of participants in each iteration who had the change in MCS scores above the MCID was 4, 4, and 3. One participant in the 1st iteration reported a reduction in the PCS and MCS scores by 14 and 33 points, respectively, due to personal contextual factors unrelated to the study.

The m6-MAT distance increased by 29.2 ± 33.7% from baseline after the initial 8 weeks of training (*n* = 10). Those who completed additional weeks of training (i.e., completing > one iteration; *n* = 5) showed similar improvement in each iteration (pre- vs. post-assessment within an iteration), but not exceeding the effects obtained from the initial 8 weeks of training (Supplementary Material 6.1). Additionally, participants increased strength of the shoulder flexors, shoulder extensors, trunk flexors, and trunk extensors by 44.5 ± 43.9%, 38.8 ± 17.8%, 49.3 ± 55.1%, and 42.9 ± 55.1%, respectively, from baseline after the initial 8 weeks of training (*n* = 7). Three participants from the Greenbank gym did not undertake the measurement of trunk muscle strength due to no suitable equipment available in the facility; one participant from the MoveWell did not attend the post-assessment. Those who completed additional weeks of training showed similar or slightly less increases in strength after each additional iteration (Supplementary Material 6.2).

## Discussion

This study co-developed and piloted a group-based ACE programme tailored for individuals with neurological impairments and limited lower-limb mobility and evaluated its feasibility in real-world community settings. The programme was well-received by participants, with high class satisfaction ratings and adherence, in particular, in the Movewell centre, and no serious adverse events. Participants successfully exercised at moderate-to-vigorous intensities, as indicated by HR monitoring and session RPE, demonstrating the feasibility of delivering a group-based structured ACE safely and effectively outside clinical settings. Preliminary outcomes suggest improvements in physical function, including fitness and strength, and self-reported quality of life. Feedback from participants further highlighted the value of the music-guided format, accessibility, and the social aspects of the group classes. Collectively, these findings support the feasibility, acceptability, and potential health benefits of implementing an ACE programme in inclusive, community-based formats.

### Exercise intensity and safety monitoring in community settings

There is growing evidence that moderate-to-vigorous intensity exercise is more effective than low-intensity training for improving physical outcomes in people with neurological conditions. Systematic reviews and clinical studies have shown that locomotor training at 60–85% of HR reserve or maximal (HR_max_) leads to greater improvements in mobility outcomes in stroke and SCI populations compared to low-intensity interventions [32, 33]. Furthermore, studies in SCI populations reported superior improvement in aerobic capacity and function following training at moderate-to-vigorous intensity (e.g., 70–80% HR reserve) [34, 35]. High-intensity training (> 85% HRmax) has shown benefits in aerobic capacity, function, and quality of life in children and adolescents with cerebral palsy [36, 37]. None of these studies reported significant adverse events. Additionally, A meta-analysis of 20 studies in stroke survivors further supports the safety and efficacy of aerobic training, while several trials of high-intensity interval training in individuals with SCI also report no serious adverse events [38].

Despite this, concerns remain around the safe delivery of high-intensity training in community settings. In our study, we adopted a comprehensive approach to safety: participants completed the PAR-Q for pre-screening, BP was monitored periodically, and volunteers were trained to interpret these readings. This approach enabled us to identify a participant with elevated blood pressure upon arrival, likely due to travel to the venue, and take appropriate measures to ensure their safety. All participants were encouraged to report health changes each week and to discuss their exercise responses with their healthcare providers. This partnership model allowed us to implement high-intensity training protocols safely and effectively in a real-world setting.

To support the fidelity of exercise intensity, we monitored RPE and HR using commercially available chest-worn monitors. These devices were easy to implement in group settings, and well tolerated by participants. Although individuals with hand impairments needed assistance to put on the monitors, they required minimal adjustment once in place. Qualitative feedback confirmed that participants found real-time heart rate monitoring feedback motivating and helpful, and many adopted wearable technology to track their heart rate outside of class. Participants consistently exercised at moderate-to-high intensity across multiple iterations with no serious adverse events, suggesting that our monitoring and safety strategies were effective. Future research with a larger sample size should examine the relationship between session CR-10 RPE and HR in people with neurological impairments. This practical approach offers a scalable model for delivering aerobic training at moderate-to-vigorous intensity to individuals with neurological impairments in community-based environments.

### Inclusivity and social connection

Our findings indicate improvements in HRQoL, including both physical and mental components, as reported by participants. Participants also highlighted psychological and social benefits from attending the group-based ACE classes. HRQoL is a multidimensional construct encompassing physical, mental, and social well-being [39], and has been shown to be negatively affected by disability and limited social participation [40–42]. Our results support the previous studies, highlighting the contribution of inclusive, group-based exercise to public health.

Participants reported additional physical benefits, such as better sleep quality, reduced muscle spasm, and weight loss, which may also contribute to enhanced HRQoL. Those benefits are likely due to regular exercise for a longer period of time [43–45]; adherence was high in all iterations of the programme. Although these additional benefits were not objectively quantified, this is the first study, to our best knowledge, showing the holistic benefits to the individuals from a community-, group-based exercise programme.

A distinctive feature of our programme was its inclusive design, allowing individuals with different neurological impairments to participate together. While condition-specific programmes allow tailored interventions, they can limit scalability and generalisability in community settings. Our approach, by contrast, used adaptive strategies such as height-adjustable tables, self-selected cadence and resistance, and HR monitoring to accommodate different mobility and fitness levels. Additional support measures, including exercise gloves, gripping aids, and strategic placement for participants with spatial neglect, were crucial in ensuring accessibility. Hence, we recommend that practitioners consult with individuals to provide appropriate logistical and functional support, enabling broader participation.

Furthermore, we used both rehabilitation-specialised (Monark 881E costing over £2,000) and commercial (for general wellbeing purposes; MagneTrainer ER costing under £200) arm ergometers, alongside height-adjustable tables ranging from £200 to £1,600. We did not observe any clear difference in participant experience or preference across equipment types. This suggests that our programme may be implemented flexibly across settings and organisations with varying budget capacities.

Our approach echoes prior work which reported factors supporting long-term adherence to exercise, such as supervision, peer support, specialist support, logistical support and motivation support [46]. Importantly, with such an inclusive approach, we were still able to demonstrate the benefits of the exercise programme through both participant-reported outcomes and objective measurements. These findings demonstrate that inclusive, group-based exercise is not only feasible but also valued by participants and can be successfully implemented in real-world community environments. Whether these benefits translate to increased physical activity outside the classes remains unknown. Future studies should include objective activity monitors to explore long-term behaviour change, as well as qualitative interviews with class leaders or trainers to capture their perspectives on programme delivery and implementation.

### Limitations

This study has several limitations. While participants achieved target exercise intensities and showed functional improvements, we were unable to confirm changes in physical function against gold-standard measures due to methodological constraints. Distance from our m6-MAT, although pragmatic, is yet to be validated against peak (e.g., peak oxygen consumption or power output) or submaximal CPET outcomes. Performing a 6-minute push test was not feasible due to space constraints and variability in wheelchair use. Whilst we standardised arm ergometers setups and resistance settings for pre- and post-tests, the reliability and validity of our adapted test require further evaluation. Additionally, due to equipment limitations, real-time HR monitoring was not available during sessions at the Greenbank gym or during the home sessions and therefore the exact exercise intensities could not be verified those settings.

The sample size was small and did not allow for inferential statistical analysis. Attendance also varied between sites, with lower adherence at the Greenbank site. Qualitative feedback suggested that scheduling conflicts contributed to reduced participation at Greenbank, and the small number of participants there likely magnified the impact of individual availability. Despite these limitations, this is the first study, to our knowledge, to deliver a long-term, inclusive, group-based ACE programme in a community setting for individuals with diverse neurological conditions. Our focus was therefore on evaluating feasibility and acceptability.

Moreover, transport and care arrangements were reported as barriers by 25% of participants, limiting attendance despite overall strong engagement. Interestingly, adherence to the home sessions was low. While these finding may appear contradictory, they reflect the complex challenges people with neurological impairments face when engaging in exercise outside structured, supervised environments. Low adherence to the home sessions may stem from difficulties maintaining motivation and structure when exercising independently, as well as the limited real-time interaction offered by the pre-recorded videos. Participants also described practical barriers to home sessions, including limited space, competing household demands, and difficulty interacting with pre-recorded videos (e.g., being unable to pause due to impaired hand function). These factors collectively likely contributed to low engagement with the home sessions. These highlight the need to explore alternative approaches, such as live online classes, greater variation in home-based programmes, opportunities for peer interaction, and accessible digital features, to better support participation in home sessions.

## Conclusions

Our findings demonstrate the feasibility and acceptability of delivering a group-based ACE programme for people with neurological impairments in community settings. Participants exercised safely at recommended intensities, reported improved HRQoL, and experienced no serious adverse events. High adherence, coupled with qualitative reports of physical, mental, and social benefits, highlights the potential for this model to address current gaps in inclusive, community-based healthcare provision. In line with the practical adaptations described (such as gripping aids, tailored positioning), incorporating condition-specific support within a generalised group format, alongside potential hybrid delivery options, may enable wider adoption and sustainability.

## Supplementary Information

Supplementary Material 1.

Supplementary Material 2.

Supplementary Material 3.

Supplementary Material 4.

Supplementary Material 5.

Supplementary Material 6.

## Data Availability

The datasets used and/or analysed during the current study are available from the corresponding author on reasonable request.
