# Supplementary material for: Co-development and implementation of a group-based arm-crank exercise programme in the community for individuals with neurological impairments
Source: BMC Sports Sci Med Rehabil. 2026 Jan 27;18:97. doi: 10.1186/s13102-025-01507-6 (PMC12917964; doi:10.1186/s13102-025-01507-6)
Supplement: Supplementary file 1 — Supplementary Material 1. [file 13102_2025_1507_MOESM1_ESM.docx]

**Supplementary material 1**

A video showing the arm-crank classes at the MoveWell: <https://www.youtube.com/watch?v=8rjXBsXopj8>
