# Supplementary material for: Co-development and implementation of a group-based arm-crank exercise programme in the community for individuals with neurological impairments
Source: BMC Sports Sci Med Rehabil. 2026 Jan 27;18:97. doi: 10.1186/s13102-025-01507-6 (PMC12917964; doi:10.1186/s13102-025-01507-6)
Supplement: Supplementary file 2 — Supplementary Material 2. [file 13102_2025_1507_MOESM2_ESM.pdf]

Supplementary materials 2: Sample ACE class routine

Bout	Song name	Track Type	Intervals<br>(1, 2, and Max indicate three different cadences)
1	Feel So Close	Speed	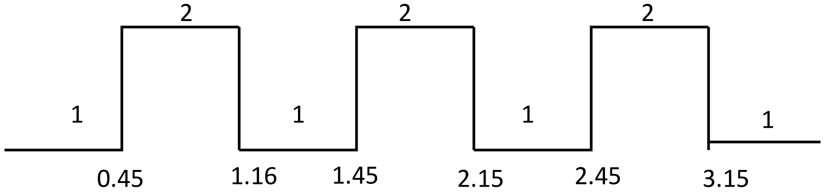
2	Physical	Strength	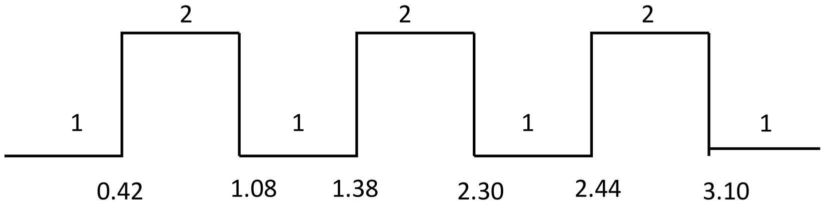
3	Good 4 U	Speed	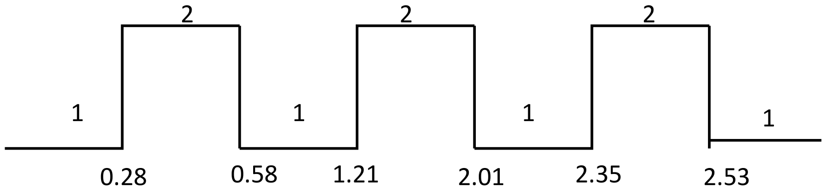

4	Song 2	Strength	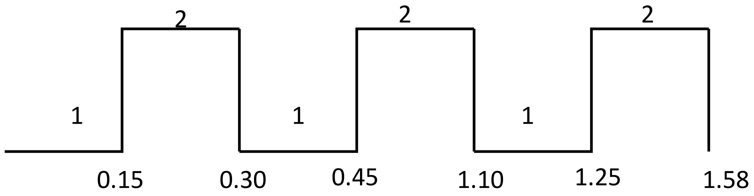
5	All the Small Things	Speed	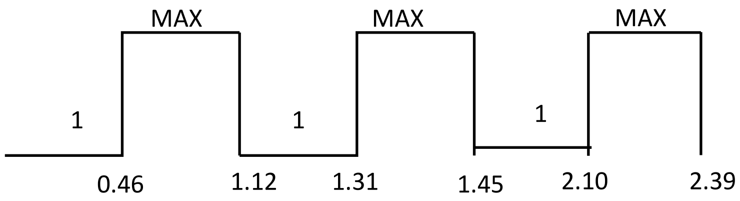
6	The Middle	Strength	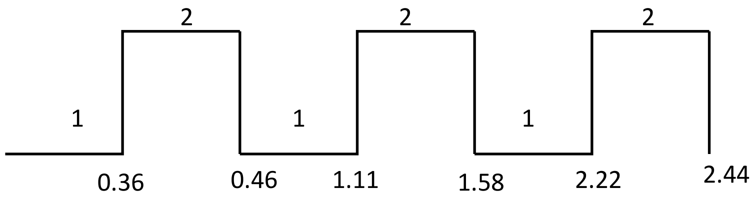
7	Everybody Talks	Speed	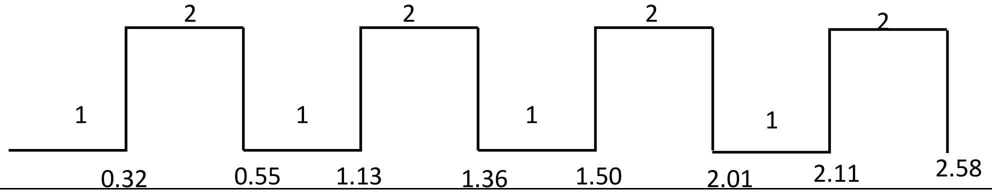
8	Rain on Me	Strength	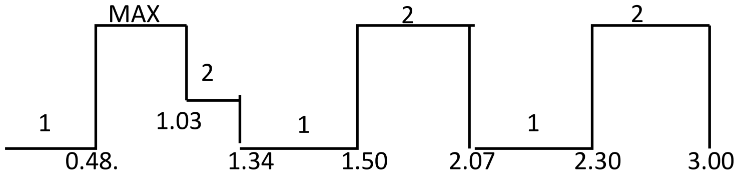

9	Shut Up and Dance	Speed	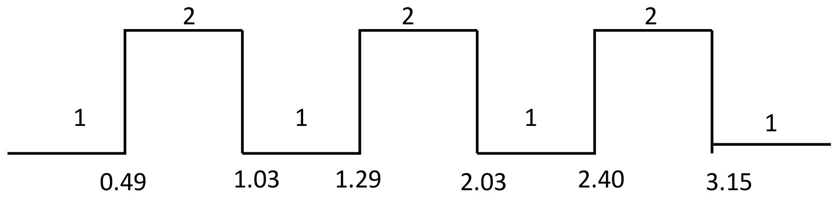
10	Easy Love Sigala	Strength	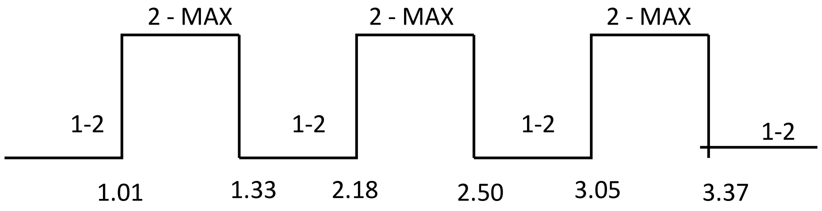
11	I predict a Riot	Strength	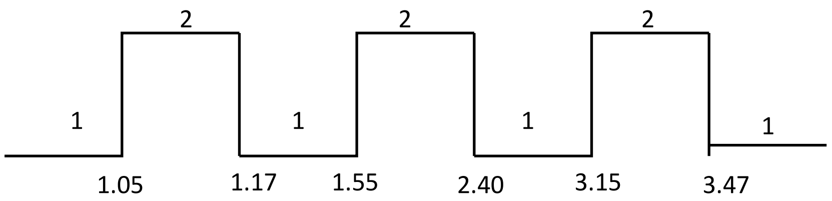
12	A sky full of stars	Speed	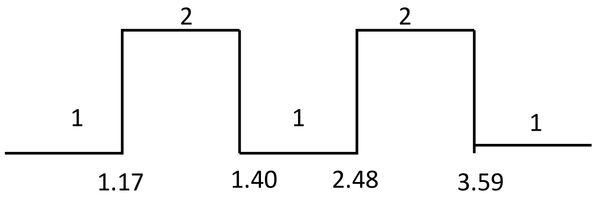
