# Supplementary material for: Co-development and implementation of a group-based arm-crank exercise programme in the community for individuals with neurological impairments
Source: BMC Sports Sci Med Rehabil. 2026 Jan 27;18:97. doi: 10.1186/s13102-025-01507-6 (PMC12917964; doi:10.1186/s13102-025-01507-6)
Supplement: Supplementary file 3 — Supplementary Material 3. [file 13102_2025_1507_MOESM3_ESM.docx]

*Cardiopulmonary exercise test (CPET)*

Participants with SCI at or above the sixth thoracic level (T6) underwent CPET to determine their peak heart rate (HR_peak_) to account for possible autonomic dysfunction, rather than relying on age-predicted peak values, which have been challenged in this specific population^1^. A standardised protocol^2^ was followed and the test was conducted using a Lode Angio CPET ergometer. Briefly, following a 5-minute warm-up, participants completed a graded exercise protocol to volitional exhaustion (defined as cadence <30 rpm on three occasions or >10 seconds on one occasion). The workload increased every 2 minutes (5 W for tetraplegia, 10 W for paraplegia). Breath-by-breath gas exchange was recorded using a Vyntus CPX metabolic cart. The highest 15-breath rolling average of V̇O_2_ was recorded as V̇O_2peak_, with the corresponding heart rate recorded as HR_peak_. V̇O_2peak_ was considered achieved if two of the following were met: (1) V̇O_2_ plateau, (2) respiratory exchange ratio ≥1.15, (3) RPE ≥19.

**References**

1 Hodgkiss, D. D. *et al.* Ergogenic effects of spinal cord stimulation on exercise performance following spinal cord injury. *Front Neurosci* **18**, 1435716 (2024). <https://doi.org/10.3389/fnins.2024.1435716>

2 Hodgkiss, D. D. *et al.* Short- and long-term effects of transcutaneous spinal cord stimulation on autonomic cardiovascular control and arm-crank exercise capacity in individuals with a spinal cord injury (STIMEX-SCI): study protocol. *BMJ Open* **15**, e089756 (2025). <https://doi.org/10.1136/bmjopen-2024-089756>
