# Supplementary material for: Co-development and implementation of a group-based arm-crank exercise programme in the community for individuals with neurological impairments
Source: BMC Sports Sci Med Rehabil. 2026 Jan 27;18:97. doi: 10.1186/s13102-025-01507-6 (PMC12917964; doi:10.1186/s13102-025-01507-6)
Supplement: Supplementary file 4 — Supplementary Material 4. [file 13102_2025_1507_MOESM4_ESM.docx]

**Arm-Crank Ergometer Spin Exercise Programme in Promoting Fitness and Physical Function After Spinal Cord Injury**

**Focus group schedule**

1. **Arm-crank ergometer spin exercise programme**
2. How have you found taking part in the arm cycling programme?
3. What have you made of the class content?
   1. Which parts do you like the most about the classes?
   2. How have you liked the difficulty?
   3. How do you feel about the strength tracks and the speed tracks?
   4. What do you think of music and class leaders?
   5. Were there additional supports or needs that could have been considered?
4. What benefits have you noticed from participating in the class?
5. What are some of the barriers to participate in for you?
   1. What have you overcome to attend classes but might still make it difficult sometimes?
   2. What are some of the reasons for missed sessions?
   3. What barriers might prevent you from participating in the future if any?
   4. Can anything be changed in the programme to reduce these barriers?
6. How have you found training at home with the arm-bike and with the online videos?
7. How could we enhance the at-home component of the programme to better motivate people to engage with it?
8. **Implementation to a wider community**
9. Based on your experience, what do you see as the next steps for this programme?
10. Who do you think would be the best providers to deliver this programme in the community?
11. How do you think this programme could be made more accessible to people who might benefit from it?
12. **Closing**
13. Is there anything else you would like to say about what you have discussed?

Thank everyone for their time and useful participation.
