# Supplementary material for: Co-development and implementation of a group-based arm-crank exercise programme in the community for individuals with neurological impairments
Source: BMC Sports Sci Med Rehabil. 2026 Jan 27;18:97. doi: 10.1186/s13102-025-01507-6 (PMC12917964; doi:10.1186/s13102-025-01507-6)
Supplement: Supplementary file 5 — Supplementary Material 5. [file 13102_2025_1507_MOESM5_ESM.docx]

**Supplementary Material 5. Focus group sample quotes.**

| Subtheme | Sample quotes |
| --- | --- |
| Theme 1: Experience of the programme | |
| *Social connection* | P1: Feeling like you are part of something.  P2: If there’s a group of us, I just work harder.  P3: I like going out to do it and speaking to and meeting people that I wouldn't.  P4: I like the group dynamic and everyone in the group.  P5: it's not just about the exercise; it's about the social interaction.  P6: I like having that structure and then I also like going out to do it as well |
| *Benefits* | P1: The fitness and pushing yourself and stuff like that.  P2: It’s not just feeling physically more fit, it’s mentally as well.  P4: I really enjoy just like moving and like exercising and like getting like my heart rate up and like feeling like I've done work because everything else is quite sedentary.  P5: My twitching during the night has got better, so I can sleep better. |
| *Enjoyment* | P1: I’ve appreciated it like. You know, the whole programme.  P4: I've thoroughly enjoyed it. I like routine  P5:I think it was fun. |
| Theme 2: Programme design and delivery | |
| *Accessibility* | P1: I like the fact that how you’ve involved people in the classes and explained what’s available.  P2: with the hand bike and the way you do it, the spin class type style. It’s really good mate, yeah.  P4: it’s really important thing for groups that don't have the luxury to go just rock up to a spin class every now and again.  P5: I haven't done exercise regularly, because it's just so hard to access. But for this (the programme) I didn't need a carer to come with me, and it was accessible.  P5: I feel really comfortable with you guys, |
| *Music* | P1: When the [music] beat kicks in and all the stuff like that as well.  P2: You’ve got an instructor-led music, I like that.  P5: I really do get carried away with the music, and then, once I'm in the Zone. I just do. I go for it.  P6: The music was always good. it was always fun. |
| *Volunteer instructors* | P4: everybody was understanding of our own individual needs.  P5: I think it's a good thing to have different people take the class because you don't get used to one person |
| *Structure* | P1: Because obviously you can’t just go 100% [strength or speed] for half an hour or an hour.  P3: It’s a good balance. Like you couldn’t just do it non-stop on the strength or doing it non-stop on the speed.  P4: What I found great was going between the 2 [alternating resistance and cadence].  P5: I find it difficult when there's like multiple speed or multiple strength in a row. The alternate was really good. They gave my body different pace to go.  P6: The good thing about varying it is before you know it, that that 50 min has gone |
| Theme 3: Challenges to programme participation | |
| *Scheduling conflicts* | P4: Friday was the more difficult day. The traffic in the city is getting worse. I had to get back to meet somebody here every Friday about 3 o'clock.  P5: It was like stuff at work; if I had meetings at the same time, I couldn't go [to the class].  P6: It's like with the Friday session. It's slap bang in the middle of the day. I'm also very busy with other bits of work. |
| *Home-based delivery barriers* | P4: I wouldn't have used the YouTube videos, mainly because once my hands are strapped to the to the actual hand bike. Then I couldn't change the channel.  P5: I couldn't take the thing home because I couldn't carry it in my bag, and I didn't have a table that I could put [the arm bike] on. |
| Theme 4: Implementation | |
| *Hybrid model* | P1: it’s always good some days if you’ve got stuff on and getting to the gym is a bit of a pain, doing all this at home is not a step down.  P2: As a package it’s sort of all those put together to be honest, yeah. Definitely. I think it’s a hybrid and it works for me.  P3: if you’re doing the lives, rather than just having the video and doing also a combination of live sessions that you can actually build into.  P4: it's nice of an online session as a backup  P5: that would be slightly better compared to just doing it by yourself.  P6: It would be nice to know, and then have maybe the option of joining in remotely because that could like for the for the days where, say I was struggling to kind of attend because of work, or something like that I probably could have from home |
| *Wider applicability* | P3: It’s not just for people in wheelchairs.  P4: having different types of neurological conditions  P5: it could be expanded to different staff members  P6: You could even potentially open it up to performance [of sports]. From my own experience of being a disabled rower and there being no fitness facilities at the club |
| *Community-based delivery* | P4: Community group that is led by with a physio or PT instructor.  P6: the community groups and charities to reach the intended target population. |

Note: The participant numbers shown in this table do not correspond to the study IDs listed in Table 1.
