# Supplementary material for: Co-development and implementation of a group-based arm-crank exercise programme in the community for individuals with neurological impairments
Source: BMC Sports Sci Med Rehabil. 2026 Jan 27;18:97. doi: 10.1186/s13102-025-01507-6 (PMC12917964; doi:10.1186/s13102-025-01507-6)
Supplement: Supplementary file 6 — Supplementary Material 6. [file 13102_2025_1507_MOESM6_ESM.pdf]

Supplementary material 6

1. **Results of short-form 36 walk-wheel questionnaire**

PCS: physical component summary; MCS: mental component summary. Data are presented as mean ± standard deviation.

	Iteration 1 (n=5)		Iteration 2 (n=8)		Iteration 3 (n=5)	
	**Pre**	**Post**	**Pre**	**Post**	**Pre**	**Post**
PCS (points)	48.4 ± 9.7	62.1 ± 25.1	48.4 ± 15.8	56.6 ± 18.9	57.3 ±18.2	64.1 ±11.4
MCS	66.7 ±11.9	76.7 ± 20.7	59.8 ± 16.2	71.3 ±19.7	75.1 ± 5.3	83.0 ± 6.5

2. **Results of physical performance and muscle strength**

	**8 weeks (n=10)**	**>8 weeks (n=5)**
**m6-MAT (% pre-assessment of iteration)**	129.3 ± 33.7	128.2 ± 16.6
**Shoulder flexors (%)**	144.5 ± 43.9	139.1 ± 12.2
**Shoulder extensors (%)**	138.8 ± 17.8	131.5 ± 24.4
**Trunk flexors (%)**	149.3 ± 55.1	125.9 ± 36.1
**Trunk extensors (%)**	142.9 ± 55.1	138.3 ± 29.9

Data are presented in mean ± standard deviation. Data from the initial 8 weeks of training as well as from additional iterations (>8 weeks) are presented separately. m6-MAT: modified 6-Minute Arm Test.
